# Supplementary material for: ClusterApp to visualize, organize, and navigate metabolomics data
Source: PLoS Comput Biol. 2026 Jul 29;22(7):e1014586. doi: 10.1371/journal.pcbi.1014586 (PMC13485123; doi:10.1371/journal.pcbi.1014586)
Supplement: Supplementary file 1 [file pcbi.1014586.s003.pdf]

Dear Editors and Reviewers,

We would like to thank you for all your effort in evaluating our manuscript. We tried to answer all queries by performing a series of new analyses, modifying the app, which is available in its modified version (http://ccbl-apps.fcfrp.usp.br/ClusterApp), providing new Jupyter Notebooks with example analyses, creating a new supplementary material, and modifying the manuscript. We hope to have answered all reviewers' queries, and we would be happy to answer any additional questions.

Sincerely,

Ricardo R. da Silva

Reviewers' comments:

Reviewer's Responses to Questions

Comments to the Authors:
Please note here if the review is uploaded as an attachment.

Reviewer #1: The manuscript presents a user-friendly and accessible web application for exploratory data analysis and visualization of user-specified metabolomics data. The development of intuitive tools for researchers without formal computational training is valuable and timely. Several aspects can further be clarified or strengthened to enhance the utility and interpretability of the framework.

1. The current figures, particularly the pixellated Emperor plots, make it difficult to clearly interpret sample clustering patterns. Improving the rendering quality or providing higher-resolution vector graphics output would enhance interpretability and visual appeal. The same consideration should apply when users download visualizations from the web application.

A1.

We thank the reviewer for this criticism. We tried to create new conversions from Emperor's svg format to a higher resolution png, which are now on the corrected manuscript version. The Emperor offers the possibility to create vectorized images that can be easily processed with open-source software, such as Inkscape. We believe the users will benefit from this feature when using ClusterApp.

2. The manuscript could further elaborate on best practices for handling sparse metabolomics datasets. It would be useful to clarify the filtering strategy for metabolites detected in negative blank controls. For datasets lacking blank control lists, the authors could discuss whether publicly available resources or libraries of common contaminant metabolites, potentially stratified by instrument type or experimental design, exist and could serve as references. This would be particularly helpful for users performing exploratory analyses where blanks were not collected.

A2.

Again, we thank the reviewer for the insightful comments. As we discussed in our manuscript, our approach for blank removal was designed to adjust the filter to sparse datasets, where the diverse nature of samples can create features that are present in only a few samples. As the reviewer points out, the sparsity can come from different origins and a complete discussion of these possibilities would require an in-depth discussion, which we believe is out of scope of this work. However, we believe that CluterApp users can benefit from different dissimilarity measures to explore sparse datasets. One sparse representation often used in metabolomics is the presence-absence matrix of MS2 spectra. If we represent the BAAT dataset used to showcase the features of ClusterApp in the main text (Figure 3) as a sparse representation (88% zeros), we see that binary-based, such as Jaccard dissimilarities, significantly improve the silhouette from 0.0003 (complete matrix) to 0.161 (sparse matrix), Supplementary Figure 3. The gnps_to_binary.ipynb was added to ClusterApp's git repository, and the main text now has additional advice to users:

An alternative approach is the use of binary dissimilarity measures for sparse datasets. ClusterApp offers different binary measures (Supplementary Table 2) that can have comparable performance to measures designed to continuous data (Supplementary Figure 3).

3. Additional guidance on the choice of distance metrics and normalization methods for different dataset characteristics would be highly beneficial. The authors could specify when total ion current (TIC) normalization, probabilistic quotient normalization (PQN), or log transformation are most appropriate, and provide recommended workflows for extremely sparse or zero-inflated datasets. Regarding the use of Bray–Curtis dissimilarity, further explanation of when this or alternative metrics (e.g., Aitchison, Euclidean) are suitable would aid users. It would be helpful to clarify whether Bray–Curtis remains reliable for highly sparse data or if there are cases where it may fail to capture meaningful biological variation.

A3.

Following the reviewer's guidance, we provide a short description accompanied by a literature reference for each measurement used (Supplementary Tables 1 and 2). As the measures we provide are limited, we believe that the GitHub repository with Jupyter notebooks can also expand the use possibilities by adding new normalization, scaling, and dissimilarity measures.

4. As methods such as t-SNE and UMAP are increasingly used for visualizing high-dimensional omics data, it would be valuable for the authors to discuss how these techniques could be integrated into their framework. The authors could also expand their visualization options by exploring existing plugins within the QIIME2 ecosystem, such as q2-umap for metabolomics datasets.

A4.

We thank the reviewer for this advice. The additions of UMAP and T-SNE have great potential. These techniques can be very valuable for complex nonlinear relationships in the data. We also observed in our tests that the projection of dissimilarity matrices improves the clustering when compared to the projection of the original features, as it is usually performed by applications of these techniques. These projection methods can now be easily selected in a separate dropdown menu in the app ('method'). The Supplementary Figures 1 and 2 now show how these projections perform, and the figures are referred to in the main text. We also

added a jupyter notebook (mainfold) with step-by-step data retrieval, transformation, and projection.

Reviewer #2: The authors have developed ClusterAPP APP, the authors did not given details about the validation of the tool. It could be better by citing one example how the developed APP is working.

A1

We thank the reviewer for the assessment of our work. We now include a supplementary file with 3 Tables and 4 Figures illustrating various validation analyses, such as comparisons between the visualization of different metrics, use of different projection methods, clustering of binary data, and a comparison of clustering performance for all dissimilarities available in the ClusterApp. The silhouette score measures how similar a sample is to its own cluster (cohesion) compared to other clusters (separation). The silhouette score ranges from −1 to +1, where a high value indicates that the object is well matched to its own cluster and poorly matched to neighboring clusters. Additionally, new Python notebooks were added to the GitHub project (https://github.com/computational-chemical-biology/ClusterApp), providing detailed information for all steps. Finally, we understand that this section in the journal is intended to describe new software. The methodological importance was demonstrated in previous publications, especially in Nat Methods. 2010 Oct;7(10):813–9. All the code to run the app locally or modify its behavior is also available; we believe that all analyses, as well as implementing strategies, are validated and available to all users. We would be happy to perform any specific validation that the reviewer requires.
