# Supplementary material for: ClusterApp to visualize, organize, and navigate metabolomics data
Source: PLoS Comput Biol. 2026 Jul 29;22(7):e1014586. doi: 10.1371/journal.pcbi.1014586 (PMC13485123; doi:10.1371/journal.pcbi.1014586)
Supplement: Supplementary file 2 [file pcbi.1014586.s001.docx]

**ClusterApp to visualize, organize, and navigate metabolomics data**

Vinicius Hansel^1$^, Pothuvilage Karunarathne^2$^, Tiago Cabral Borelli^1^, Isabela Victorino da Silva Amatto^1^, Matheus de Lima Ortega^1^, Robert Quinn^2*^, Ricardo R. da Silva^1*^

1. Department of Biomolecular Sciences, Computational Chemical Biology Laboratory, School of Pharmaceutical Sciences of Ribeirão Preto, University of São Paulo, Ribeirão Preto, Brazil

2. Department of Biochemistry and Molecular Biology, Michigan State University, East Lansing, USA

$Contributed equally

* Corresponding authors

RR da Silva - [ridasilva@usp.br](mailto:ridasilva@usp.br)

R Quinn - [quinnrob@msu.edu](mailto:quinnrob@msu.edu)

Table A - Description of normalization methods available through ClusterApp.

| Type of Normalization | Formula | Description |
| --- | --- | --- |
| Total Ion Current^[1]^ |  | Total Ion Current (TIC) normalization divides and scales each feature by the total signal in a sample, converting absolute intensities into relative abundances. This method assumes that variations in total signal are due solely to differences in sample concentration or injection amount, which may not be true when individual features change independently. |
| Probabilistic Quotient^1^ |  | Probabilistic Quotient Normalization (PQN) corrects for overall differences in sample concentration by scaling each sample using the median of feature ratios relative to a reference spectrum. This approach is robust to features that change independently, making it suitable for spectra where only part of the profile varies. |

Table B - Description of dissimilarity metric available through ClusterApp.

| Type of Dissimilarity Metric | Formula | Description |
| --- | --- | --- |
| Canberra^[2]^ |  | Canberra distance is a metric that measures the dissimilarity between two points in a multidimensional space by calculating the ratios of the differences of their corresponding coordinates to the sum of their absolute values. This makes it particularly sensitive to differences in features with smaller magnitudes. |
| Bray-Curtis^[3]^ |  | Bray–Curtis dissimilarity quantifies the difference between two samples based on abundances of observed features. It ranges from 0 (identical compositions) to 1 (completely disjoint compositions). It is widely used in ecology, microbiome studies, and any context where relative abundances matter. |
| Euclidean^3^ |  | The Euclidean distance is the standard metric of straight-line distance between two points in a multidimensional space. It is sensitive to absolute magnitudes and requires proper normalization when comparing samples with different total intensities. |
| Standardized Euclidean^[4]^ |  | The standardized Euclidean distance is a metric that measures the distance between two multivariate points while accounting for differences in scale or variability across variables. Unlike ordinary Euclidean distance, each variable is scaled by its variance, so that variables with larger variability do not dominate the distance computation. |
| City Block/Manhattan^[5]^ |  | Manhattan distance is a metric that measures the distance between two points in a multidimensional space by calculating the differences of their corresponding coordinates, rather than measuring the distance in a straight line. Unlike Euclidean distance, it does not exaggerate the influence of large coordinate differences due to squaring. |
| Chebyshev^[6]^ |  | Chebyshev distance is a metric that measures the distance between two points in a multidimensional space by taking the largest difference among their corresponding coordinates, making it useful in applications where the maximum deviation dominates the measure of dissimilarity. |
| Minkowski^[7]^ |  | Minkowski distance is a general metric that generalizes several common distance metrics by introducing a parameter *p* that controls the emphasis on differences across features. This flexibility allows the metric to emphasize either large or small differences across features, depending on the choice of *p*. |
| Correlation^[8]^ |  | Correlation dissimilarity is a metric that quantifies the degree of similarity in the patterns of features between two samples, independent of their absolute magnitudes. It measures how linearly related the features of two samples are, where a value of 0 indicates perfectly correlated feature patterns, and a value of 1 indicates completely uncorrelated patterns (or perfectly anti-correlated patterns, if negative correlations are considered), being best for capturing relative trends. |
| Cosine^[9]^ |  | Cosine dissimilarity measures the difference in orientation between two vectors in multidimensional space, focusing on the angle between them rather than their absolute magnitudes, where a value of 0 indicates that the vectors point in the same direction (perfectly aligned patterns) and a value of 1 indicates that the vectors are orthogonal (completely dissimilar patterns). Unlike Pearson correlation, cosine dissimilarity is sensitive to shifts: adding a constant to all elements of a vector alters the cosine dissimilarity, but it is scale-invariant: multiplying all elements of a vector by a positive constant does not change the metric. |
| Dice^[10]^ |   Where:   - a: number of shared presences (1–1 matches) - b: number of features present in x but absent in y (1-0 matches) - c: number of features absent in x but present in y (0-1 matches) | The Dice dissimilarity quantifies the difference between two binary vectors by comparing their shared presences relative to the total number of presences in both vectors, giving double weight to simultaneous presences. This metric is particularly appropriate for sparse binary data where shared activations are more informative than shared absences, and where the intensity or magnitude of features is not considered. |
| Hamming^[11]^ |   Where:   - b: number of features present in x but absent in y (1-0 matches) - c: number of features absent in x but present in y (0-1 matches) - p=a+b+c+d: total number of variables | The Hamming distance metric quantifies the difference between two vectors by measuring the proportion of positions at which the corresponding elements differ, treating both shared presences and shared absences are treated as agreements, while any inequality contributes identically to the total distance. This metric is particularly appropriate for discrete or categorical data where features are aligned and the primary interest is structural disagreement rather than co-occurrence patterns or magnitude effects, making it insensitive to sparsity or imbalance in feature activation. |
| Jaccard^[12]^ |   Where:   - a: number of shared presences (1–1 matches) - b: number of features present in x but absent in y (1-0 matches) - c: number of features absent in x but present in y (0-1 matches) | The Jaccard dissimilarity metric quantifies the difference between two binary vectors by measuring the proportion of non-shared presences relative to the total number of features present in at least one vector. This metric is appropriate for categorical data with non-negative features and similarity better measured as shared occurrence rather than shared absence or magnitude. |
| Kulsinski^[13]^ |   Where:   - a: number of shared presences (1–1 matches) - b: number of features present in x but absent in y (1-0 matches) - c: number of features absent in x but present in y (0-1 matches) - p=a+b+c+d: total number of variables | The Kulsinski dissimilarity is a metric for comparing large binary vectors of absences and presences of variables, particularly in settings where the data are sparse and where the presence of a feature carries more information than its absence. This measure is well suited to high-dimensional binary data, as it avoids overstating similarity due to shared zeros while preserving sensitivity to informative co-occurrences. |
| Mahalanobis^[14]^ |  | The Mahalanobis metric measures the distance between an observation and a reference point while explicitly accounting for correlations among variables and differences in scale. It rescales the space so that correlated directions are compressed and low-variance directions are expanded, making it so that distance reflects how unusual an observation is relative to the joint distribution measured in number of standard deviations rather than in raw coordinate differences. |
| Matching |   Where:   - a: number of shared presences (1–1 matches) - d: number of shared absences (0–0 matches) - p=a+b+c+d: total number of variables | Defined as synonym for Hamming in SciPy’s documentation. |
| Rogers-Tanimoto^[15]^ |   Where:   - a: number of shared presences (1–1 matches) - b: number of features present in x but absent in y (1-0 matches) - c: number of features absent in x but present in y (0-1 matches) - d: number of shared absences (0–0 matches) | The Rogers–Tanimoto dissimilarity is a binary distance measure used to compare two observations described by presence–absence features. It is based on a 2×2 contingency table that counts agreements and disagreements across features. Unlike Jaccard, this is a binary dissimilarity measure that gives greater weight to mismatches between observations. |
| Russel-Rao^[16]^ | Where:   - a: number of shared presences (1–1 matches) - p: total number of variables | The Russell–Rao dissimilarity metric quantifies the difference between two binary vectors by only evaluating how much both objects simultaneously exhibit feature presence across a fixed set of attributes. This metric is particularly appropriate for sparse binary data where absolute co-occurrence of active features is the only meaningful signal, and where absence does not carry informative weight. |
| Sokal-Michener | Where:   - a: number of shared presences (1–1 matches) - b: number of features present in x but absent in y (1-0 matches) - c: number of features absent in x but present in y (0-1 matches) - d: number of shared absences (0–0 matches) | Defined as synonym for Matching in SciPy’s documentation. |
| Sokal-Sneath^[17]^ | Where:   - a: number of shared presences (1–1 matches) - b: number of features present in x but absent in y (1-0 matches) - c: number of features absent in x but present in y (0-1 matches) | The Sokal–Sneath dissimilarity metric quantifies the difference between two binary vectors by evaluating how frequently features are not simultaneously present, while only explicitly rewarding shared presences and ignoring shared absences. This metric is particularly appropriate for binary data where co-occurring active features are meaningful signals, mismatches between feature states should be penalized more strongly than in symmetric matching schemes, and joint absences do not carry informative weight. |
| Yule^[18]^ | Where:   - a: number of shared presences (1–1 matches) - b: number of features present in x but absent in y (1-0 matches) - c: number of features absent in x but present in y (0-1 matches) - d: number of shared absences (0–0 matches) | The Yule dissimilarity metric quantifies the difference between two binary vectors by comparing the balance between concordant and discordant feature pairings within a 2×2 contingency table. It is derived from Yule’s association framework and emphasizes how strongly features disagree compared to how much they agree or disagree overall. This metric is particularly appropriate for binary data where agreement and disagreement are treated as statistical dependence rather than just matching features, making it sensitive to how the variables are related rather than just whether they match. |

Figure A - t-SNE dimension reduction of BAAT dataset. A) Feature’s area under the curves. B) t-SNE dimension reduction of dissimilarity with the Bray-Curtis metric with dimension set to 2. C) t-SNE dimension reduction of dissimilarity with the Bray-Curtis metric with dimension set to 3, showing an example of the ‘crowding problem’^[19]^.

Figure B - UMAP dimension reduction of BAAT dataset. A) UMAP of Feature’s area under the curves. B) UMAP of dissimilarity with the Canberra metric with dimension set to 3.

Table C - Silhouette score^1^ calculation comparing euclidean to other dissimilarity metrics available through ClusterApp.

|  | **Mouse tissue samples** | | **Coral life history stages** | |
| --- | --- | --- | --- | --- |
| **Dissimilarity^2^** | **Silhouette score** | **Sihlouette Agglomerative** | **Silhouette score** | **Sihlouette Agglomerative** |
| canberra | 0.1085 | 0.4331 | 0.0763 | 0.2798 |
| braycurtis | 0.2029 | 0.2364 | 0.1416 | 0.2125 |
| euclidean | 0.0851 | 0.4132 | -0.1045 | 0.4348 |
| seuclidean | -0.0502 | 0.4446 | -0.0845 | 0.4324 |
| cityblock | 0.1407 | 0.4214 | -0.0471 | 0.3215 |
| chebyshev | 0.0393 | 0.3395 | -0.1266 | 0.4535 |
| correlation | 0.0393 | 0.3698 | 0.1305 | 0.3366 |
| cosine | 0.2148 | 0.3697 | 0.1313 | 0.3368 |
| hamming | -0.1206 | 0.4154 | -0.1016 | 0.2336 |
| jaccard | 0.0003 | 0.0003 | 0.0000 | 0.0000 |
| matching | -0.1206 | 0.4154 | -0.1016 | 0.2336 |
| rogerstanimoto | 0.1909 | 0.3243 | 0.1231 | 0.2634 |
| russellrao | 0.0093 | 0.0395 | 0.0150 | 0.0622 |
| sokalmichener | 0.1909 | 0.3243 | 0.1231 | 0.2634 |
| sokalsneath | 0.1026 | 0.1215 | 0.1105 | 0.1665 |
| yule | 0.3998 | 0.3365 | 0.1992 | 0.4774 |

1 - The silhouette score measures how similar a sample is to its own cluster (cohesion) compared to other clusters (separation). The silhouette score ranges from −1 to +1, where a high value indicates that the object is well matched to its own cluster and poorly matched to neighboring clusters. 2 - The metrics seuclidean, wminkowski, dice and mahalanobis were incompatible with the datasets evaluated.

Figure C - Comparison between dissimilarities performed on binary data. A) Bray-Curtis, silhouette score 0.216. B) Jaccard, silhouette score 0.161.

Figure D - Comparison between high silhouette scoring dissimilarities. A) Bray-Curtis, after blank removal, TIC normalization, and Autoscaling. B) Yule, after blank removal, TIC normalization (Autoscaling incompatible).

References

[“8 Peak Detection.” *Data Handling in Science and Technology* 21 (January 1998): 183–90. https://doi.org/10.1016/S0922-3487(98)80027-0.](https://www.zotero.org/google-docs/?D1KJ1O)

[Everitt, Brian S., Sabine Landau, Morven Leese, and Daniel Stahl. *Cluster Analysis*. John Wiley & Sons, 2011.](https://www.zotero.org/google-docs/?D1KJ1O)

[Kaufman, Leonard, and Peter J. Rousseeuw. *Finding Groups in Data: An Introduction to Cluster Analysis*. John Wiley & Sons, 2009.](https://www.zotero.org/google-docs/?D1KJ1O)

[Levy, Avivit, B. Riva Shalom, and Michal Chalamish. “A Guide to Similarity Measures and Their Data Science Applications.” *Journal of Big Data* 12, no. 1 (2025): 188. https://doi.org/10.1186/s40537-025-01227-1.](https://www.zotero.org/google-docs/?D1KJ1O)

[Maaten, Laurens van der, and Geoffrey Hinton. “Visualizing Data Using T-SNE.” *Journal of Machine Learning Research* 9, no. Nov (2008): 2579–605.](https://www.zotero.org/google-docs/?D1KJ1O)

[“Metrics, Similarity, and Sets.” https://doi.org/10.1016/B978-0-12-804452-0.00002-6.](https://www.zotero.org/google-docs/?D1KJ1O)

[*Numerical Ecology*. 2012. https://shop.elsevier.com/books/numerical-ecology/legendre/978-0-444-53868-0.](https://www.zotero.org/google-docs/?D1KJ1O)

[Ricotta, C., and J. Podani. “On Some Properties of the Bray-Curtis Dissimilarity and Their Ecological Meaning.” *Ecological Complexity* 31 (September 2017): 201–5. https://doi.org/10.1016/j.ecocom.2017.07.003.](https://www.zotero.org/google-docs/?D1KJ1O)

[ScienceDirect. “Data Mining: Concepts and Techniques.” Accessed June 1, 2026. http://www.sciencedirect.com:5070/book/monograph/9780123814791/data-mining-concepts-and-techniques.](https://www.zotero.org/google-docs/?D1KJ1O)

[“Scipy.Spatial.Distance.Kulsinski — SciPy v1.10.0 Manual.” Accessed June 1, 2026. https://docs.scipy.org/doc/scipy-1.10.0/reference/generated/scipy.spatial.distance.kulsinski.html.](https://www.zotero.org/google-docs/?D1KJ1O)

[Sun, Jun, and Yinglin Xia. “Pretreating and Normalizing Metabolomics Data for Statistical Analysis.” *Genes & Diseases* 11, no. 3 (2023): 100979. https://doi.org/10.1016/j.gendis.2023.04.018.](https://www.zotero.org/google-docs/?D1KJ1O)

[“Yule — SciPy v1.17.0 Manual.” Accessed June 1, 2026. https://docs.scipy.org/doc/scipy/reference/generated/scipy.spatial.distance.yule.html.](https://www.zotero.org/google-docs/?D1KJ1O)

1. [Sun and Xia, “Pretreating and Normalizing Metabolomics Data for Statistical Analysis.”](https://www.zotero.org/google-docs/?0Pxp6D)
2. [Everitt et al., *Cluster Analysis*.](https://www.zotero.org/google-docs/?XIkSHX)
3. [Ricotta and Podani, “On Some Properties of the Bray-Curtis Dissimilarity and Their Ecological Meaning.”](https://www.zotero.org/google-docs/?KqnElk)
4. [“8 Peak Detection.”](https://www.zotero.org/google-docs/?xgSQIf)
5. [Everitt et al., *Cluster Analysis*.](https://www.zotero.org/google-docs/?r8wnY7)
6. [“Metrics, Similarity, and Sets.”](https://www.zotero.org/google-docs/?Ka0MNG)
7. [Everitt et al., *Cluster Analysis*.](https://www.zotero.org/google-docs/?3mnWDS)
8. [Kaufman and Rousseeuw, *Finding Groups in Data*.](https://www.zotero.org/google-docs/?io8RYS)
9. [ScienceDirect, “Data Mining.”](https://www.zotero.org/google-docs/?2FMPfR)
10. [Levy et al., “A Guide to Similarity Measures and Their Data Science Applications.”](https://www.zotero.org/google-docs/?GkjP5k)
11. [Levy et al., “A Guide to Similarity Measures and Their Data Science Applications.”](https://www.zotero.org/google-docs/?NHADDn)
12. [Levy et al., “A Guide to Similarity Measures and Their Data Science Applications.”](https://www.zotero.org/google-docs/?Kz0ktg)
13. [“Scipy.Spatial.Distance.Kulsinski — SciPy v1.10.0 Manual.”](https://www.zotero.org/google-docs/?VFSeuw)
14. [Levy et al., “A Guide to Similarity Measures and Their Data Science Applications.”](https://www.zotero.org/google-docs/?UP0hae)
15. [*Numerical Ecology*.](https://www.zotero.org/google-docs/?tQJL36)
16. [*Numerical Ecology*.](https://www.zotero.org/google-docs/?cKOR56)
17. [*Numerical Ecology*.](https://www.zotero.org/google-docs/?UVE9r9)
18. [“Yule — SciPy v1.17.0 Manual.”](https://www.zotero.org/google-docs/?QbKf5y)
19. [Maaten and Hinton, “Visualizing Data Using T-SNE.”](https://www.zotero.org/google-docs/?3VuoED)
